# Scanning Micro-Mirror with an Electrostatic Spring for Compensation of Hard-Spring Nonlinearity

**DOI:** 10.3390/mi8080240

**Published:** 2017-08-04

**Authors:** Takashi Izawa, Takashi Sasaki, Kazuhiro Hane

**Affiliations:** Department of Finemechanics, Tohoku University, Aramaki-aza Aoba 6-6-01, Aoba-ku, Sendai 980-8579, Japan; takashi.izawa@aisin.co.jp (T.I.); t_sasaki@hane.mech.tohoku.ac.jp (T.S.)

**Keywords:** scanning micro mirror, nonlinear spring, resonant vibration, microelectromechanical systems

## Abstract

A scanning micro-mirror operated at the mechanical resonant frequency often suffer nonlinearity of the torsion-bar spring. The torsion-bar spring becomes harder than the linear spring with the increase of the rotation angle (hard-spring effect). The hard-spring effect of the torsion-bar spring generates several problems, such as hysteresis, frequency shift, and instability by oscillation jump. In this paper, a scanning micro-mirror with an electrostatic-comb spring is studied for compensation of the hard-spring effect of the torsion-bar spring. The hard-spring effect of the torsion-bar spring is compensated with the equivalent soft-spring effect of the electrostatic-comb spring. The oscillation curve becomes symmetric at the resonant frequency although the resonant frequency increases. Theoretical analysis is given for roughly explaining the compensation. A 0.5 mm square scanning micro-mirror having two kinds of combs, i.e., an actuator comb and a compensation comb, is fabricated from a silicon-on-insulator wafer for testing the compensation of the hard-spring in a vacuum and in atmospheric air. The bending of the oscillation curve is compensated by applying a DC voltage to the electrostatic-comb spring in vacuum and atmosphere. The compensation is attributed by theoretical approach to the soft-spring effect of the electrostatic-comb spring.

## 1. Introduction

A scanning micro-mirror is one of the key devices of micro-electro-mechanical systems. A scanning micro-mirror is a fundamental component of laser projection displays, which simply consists of a two-dimensional scanning micro-mirror and a collimated laser beam. A scanning micro-mirror is also promising for laser reflectometry. The reflected laser light from scanned object is detected for visualizing the object. Distance measurement by laser scanning by utilizing a scanning micro-mirror is much awaited key technology of automatic operation of automotive car [1]. The requirements and progresses on scanning micro-mirror are extensively reviewed [2], where several operational characteristics of micro-mirrors are described in detail. For the above purposes, wider and faster scanning is needed. In the case of a scanning micro-mirror operated at wide angle and high frequency, the mechanical resonance of torsional oscillation is often used to increase the rotational angle and to minimize the necessary force and energy for oscillational scanning. The oscillation curve is the oscillational amplitude plotted as a function of the frequency of the applied AC voltage. The oscillation curve is usually symmetric with respect to the peak resonant frequency when the scanning angle is small. By increasing the oscillation amplitude, i.e., rotation angle, the torsional spring becomes harder than the linear spring as a function of rotation, which is called the hard-spring effect [3,4]. The hard-spring effect generates a shift and a bending of the oscillation curve towards higher frequencies, which often causes instability of operation, such as hysteresis phenomenon of the oscillation curve. On the other hand, it is noteworthy from the bending of the oscillation curve that the electrostatic vertical comb for rotation shows a soft-spring property when the vertical comb does not have the height offset between the fixed and movable combs [5,6].

Resonant frequency tuning of mechanical resonators are often needed to compensate the frequency shift caused by the fabrication error and environmental changes, such as temperature. Several methods for tuning the resonant frequency were studied on the basis of electrostatic force [7,8,9,10,11,12,13] and thermal expansion [14]. In case of the electrostatic method, the effective springs by electrostatic force were incorporated in the laterally oscillating resonators [7,8,9,10,11,12]. The capacitance of the electrostatic springs was modulated as a function of displacement to generate the linear springs. The nonlinearity of a laterally-oscillating resonator was also tuned by applying a DC voltage [15]. On the other hand, the torsional resonant frequency of scanning micro-mirror was also tuned by applying a DC voltage to the electrostatic vertical combs [13]. The electrostatic vertical combs also function as an additional spring to the mechanical torsion-bar spring. However, there are few reports on the tuning of the nonlinearity of the torsion-bar spring. The thermal expansion of a Y-shaped torsional spring adjusted the tension of the spring for the parametric operation [16]. In the case of the electrostatic method, there is no report for the compensation of the hard-spring effect of the torsion-bar spring.

In this report, a method for compensating a hard-spring effect of the torsion-bar spring of the scanning micro-mirror is proposed by using the electrostatic vertical combs without a height offset between the movable and fixed combs. The hard-spring effect of the torsion-bar spring is compensated by applying a DC voltage to the electrostatic combs. An analytical model is given on the basis of variable capacitance of vertical comb electrodes for a rough explanation of the compensation of the hard-spring effect. A 0.5 mm square scanning micro-mirror is designed and fabricated from a silicon-on-insulator wafer. The oscillation curve is measured by varying the DC voltage applied to the electrostatic combs around the resonant frequency. The hard-spring effect is compensated by the applied DC voltage.

## 2. Principle

**Figure 1:**
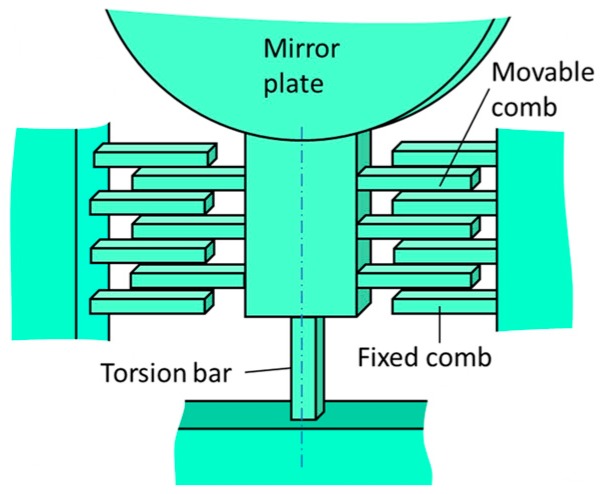
Schematic diagram of torsion-bar with electrostatic spring consisting of comb electrodes.

Figure 1 shows a part of the rotational spring of the proposed scanning micro-mirror. The mirror is supported by two silicon torsion-bars, one of which is shown in Figure 1. In addition to the silicon torsion-bar, the electrostatic vertical combs are also fabricated between the mirror plate and the silicon torsion-bar as shown in Figure 1. The torsion-bar works as a rotational spring for the rotation of the mirror plate. The rotational angle *θ* of the torsion-bar is proportional to the applied torque and the proportionality constant (i.e., the spring constant of the torsion bar) is given by *k_m_*_0_. If a nonlinearity of the torsion-bar exists at a large rotation angle, the spring constant is expressed by [17]:

(1) $$k_{m}=k_{m0}(1+\alpha \theta^{2})$$

Here, *α* is the nonlinearity coefficient. When *α* is positive, the torsion bar is a hard-spring, the oscillation amplitude, measured as a function of frequency around a rotational resonant frequency (oscillation curve), bends toward the higher frequency. When *α* is negative, it becomes a soft-spring. In the conventional torsion-bar, having a rectangular cross-section, the hard-spring effect is generated due to axial tension and is often observed at a large oscillation amplitude.

**Figure 2:**
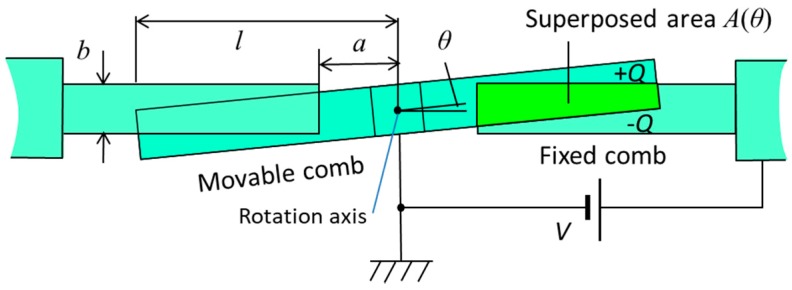
Schematic diagram of the cross-section of the electrostatic spring consisting of comb electrodes.

Here we consider the electrostatic combs as a spring, where a DC voltage is applied to the combs. In order to obtain the symmetry of spring at rotation angle ±*θ*, there is no offset for the rotational axis from the central plane of the fixed combs. The cross-sectional structure of the electrostatic combs is shown in Figure 2. Therefore, the torque generated by applying a DC voltage is always a resorting torque as a function of rotation angle. 

The energy stored in the comb electrodes is the electrostatic energy given by:

(2) $$U_{e}=\frac{CV^{2}}{2}=\frac{\varepsilon_{0}V^{2}N}{2g}A(\theta )$$

where *C* is the capacitance of the combs and *V* is the applied DC voltage. Using the overlapped area *A*(*θ*) of facing comb fingers shown in Figure 2, the capacitance *C* is expressed by *C* = *Nε*_0_*A(θ)*/*g*, where *ε*_0_ is the permittivity of vacuum, *N* is number of comb finger gaps, and *g* is the gap between the facing comb fingers. Then, the restoring torque is given by differentiating the stored energy *U_e_* as shown in Equation (3):

(3) $$T_{e}=-\frac{\partial U_{e}}{\partial \theta }=-\frac{1}{2}N\varepsilon_{0}\frac{V^{2}}{g}\frac{\partial A}{\partial \theta }$$

The electrostatic spring also shows a nonlinear effect. The simplest expression of the spring constant *k_e_* of electrostatic spring contains the lowest nonlinear coefficient *β* by neglecting the higher order terms as:

(4) $$k_{e}=k_{e0}(1-\beta \theta^{2})$$

If the nonlinear term *k_e_*_0_*β* of Equation (4) can be equal to the nonlinear term *k_m_*_0_*α* of Equation (1), the nonlinear effect of total spring is apparently compensated and the oscillation curve becomes symmetric at the resonant frequency neglecting the higher order components. When the spring constant of the electrostatic spring is given by Equation (4), then the torque *T_e_* of the electrostatic spring can be calculated by multiplying the spring constant by rotation angle as follows:

(5) $$T_{e}=k_{e0}(1-\beta \theta^{2})\theta$$

where the torque is expressed as a cubic equation of *θ*.

Using the dimension parameters of the comb fingers as shown in Figure 2, the superposed area *A*(*θ*) of the comb fingers is expressed approximately as:

(6) $$A(\theta )=\{\begin{array}{c} b(l-a)-\frac{1}{2}(l^{2}-a^{2})\theta \;(0<\theta <\frac{b}{l})\\ \frac{{\left(b-a\theta \right)}^{2}}{2\theta }\;(\frac{b}{l}<\theta <\frac{b}{a}) \end{array}$$

**Figure 3:**
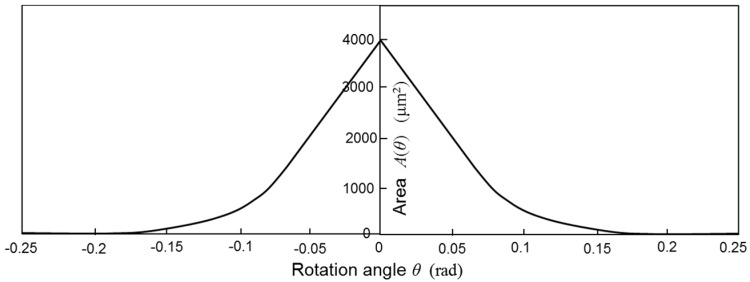
Superposed area of comb fingers as a function of rotation angle.

**Figure 4:**
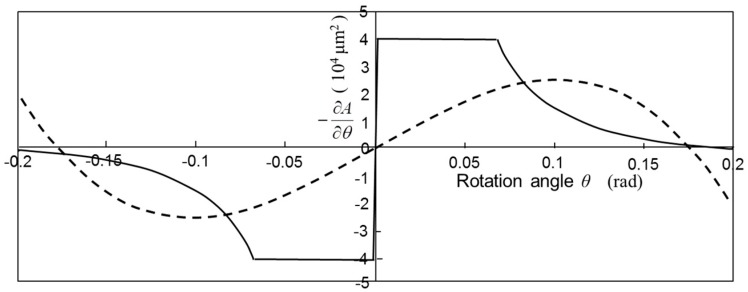
Derivation of the superposed area of comb fingers as a function of rotation angle (solid curve) and the curve by the least-square method (dotted curve).

The area *A*(*θ*) is an even function of *θ* and decreases with the increase in *θ*. Figure 3 shows the area *A*(*θ*) using the dimensions of the designed scanning micro-mirror described later in this paper. The symbols *l*, *a*, *b*, and *g* are the distance between the rotation axis and the end of movable comb finger, the distance from the rotation axis to the end of fixed comb finger, thickness of comb fingers, and gap between the fingers of movable and fixed combs. Torque *T_e_* is obtained by differentiating the stored energy of comb capacitors, and thus proportional to the differential of area as shown in Equation (3). Figure 4 shows the calculated values –*∂A*/*∂θ* as a function of *θ* using the designed dimensions of the fabricated scanning micro-mirror.

In order to obtain a rough estimation, Equation (5) may approximate the calculated values of the torque proportional to –*∂A*/*∂θ*. The least-square method is applied to obtain the approximate equation. In this method, the equation:

(7) $$Z=\int_{0}^{\theta_{m}}{\left(\frac{-1}{2}N\varepsilon_{0}\frac{V^{2}}{g}\frac{\partial A}{\partial \theta }-k_{e0}(1-\beta \theta^{2})\theta \right)}^{2}d\theta$$

is minimized to obtain the coefficients *k_e_*_0_ and *β.* The maximum angle of mechanical rotation is given by *θ_m_*. For a simple case, the maximum angle is assumed to be the angle where the overlapped area *A*(*θ*) becomes zero. When the value of *b* is much smaller than *a* and *l*, the angle *θ_m_* can be expressed as *θ_m_ =* tan*^–1^(b*/*a) ≈ b*/*a*. In addition, the angle where the shape of the overlapped area changes from the triangle to the quadrangle with the increase of rotation angle can also approximate to tan*^–1^(b*/*l) ≈ b*/*l*. The torque approximate to a third order equation is given by:

(8) $$T_{e}=\frac{1}{2}N\varepsilon_{0}\frac{V^{2}}{g}\cdot \frac{15a^{3}}{32b}\left\{\left(20\ln\frac{l}{a}-7\left(1-\frac{a^{2}}{l^{2}}\right)\right)-\frac{7a^{2}}{3b^{2}}\left(12\ln\frac{l}{a}-5\left(1-\frac{a^{2}}{l^{2}}\right)\right)\theta^{2}\right\}\theta$$

Therefore, the coefficients *k_e_*_0_ and *k_e_*_0_*β* are given, respectively, as:

(9) $$k_{e0}=\frac{1}{2}N\varepsilon_{0}\frac{V^{2}}{g}\cdot \frac{15a^{3}}{32b}\left(20\ln\frac{l}{a}-7\left(1-\frac{a^{2}}{l^{2}}\right)\right)$$

(10) $$k_{e0}\beta =\frac{1}{2}N\varepsilon_{0}\frac{V^{2}}{g}\cdot \frac{35a^{5}}{32b^{3}}\left(12\ln\frac{l}{a}-5\left(1-\frac{a^{2}}{l^{2}}\right)\right)$$

The torque approximate to Equation (8) is shown by the dotted curve in Figure 4, which shows a large nonlinearity due to the strong angle dependence of the capacitance.

When the scanner is driven periodically by an external torque *T*_0_, the motion equation is expressed as follows:

(11) $$I_{\theta }\frac{d^{2}\theta }{dt^{2}}+\gamma_{\theta }\frac{d\theta }{dt}+k_{m0}(1+\alpha \theta^{2})\theta +k_{e0}(1-\beta \theta^{2})\theta =T_{0}\sin(\omega \;t+\phi )$$

Here, *I_θ_* and *γ_θ_* are the rotational inertia and the dumping coefficient of scanning micro-mirror, and *ω* is the angular frequency of the external torque. Since the nonlinearity of electrostatic combs are considered to be soft-springs, if the hard-spring effect of the torsion bars is compensated by the soft-spring effect of the electrostatic comb, then the hard-spring nonlinearity of the scanning micro-mirror can be suppressed by the compensated condition. The compensated condition is given by equalizing the nonlinear coefficients:

(12) $$k_{m0}\alpha =k_{e0}\beta =\frac{1}{2}N\varepsilon_{0}\frac{V^{2}}{g}\cdot \frac{35a^{5}}{32b^{3}}\left(12\ln\frac{l}{a}-5\left(1-\frac{a^{2}}{l^{2}}\right)\right)$$

In addition, the equivalent spring constant of the system is increased by the addition of the electrostatic spring as expressed by the following approximate equation:

(13) $$k_{m0}+k_{e0}=k_{m0}+\frac{1}{2}N\varepsilon_{0}\frac{V^{2}}{g}\cdot \frac{15a^{3}}{32b}\left(20\ln\frac{l}{a}-7\left(1-\frac{a^{2}}{l^{2}}\right)\right)$$

On the other hand, the spring constant of the torsion-bar having a square cross-section is given by:

(14) $$k_{m0}=\frac{Ewb^{3}}{3(1-\nu )L}\left\{1-\frac{192w}{\pi^{5}b}\tanh\left(\frac{\pi b}{2w}\right)\right\}$$

where *E* is the Young’s modulus of silicon and *ν* is the Poisson ratio. The symbols *L*, *w*, and *b* represent the length, width, and thickness of torsion-bars, respectively. When the mirror rotates, the torsion of the bar generates a tension in the bar. Due to the tension, the spring constant of the torsion-bar increases (i.e., hard-spring effect), and the increase of the spring constant is expressed by [18]:

(15) $$\Delta k_{m}=k_{m0}\alpha \theta^{2}=\frac{E}{16L^{3}}\left(\frac{w^{5}b}{10}+\frac{w^{3}b^{3}}{9}+\frac{wb^{5}}{10}\right)\theta^{2}$$

Therefore, considering the hard-spring effect, the spring constant of the torsion-bar is given by:

(16) $$k_{m}=k_{m0}(1+\alpha \theta^{2})=\frac{Ewb^{3}}{3(1-\nu )L}\left\{1-\frac{192w}{\pi^{5}b}\tanh\left(\frac{\pi b}{2w}\right)\right\}+\frac{E}{16L^{3}}\left(\frac{w^{5}b}{10}+\frac{w^{3}b^{3}}{9}+\frac{wb^{5}}{10}\right)\theta^{2}$$

Using the design parameters, the values of the analytical equations are obtained. It is assumed that *L* = 200 μm, *w* = 20 μm, and *b* = 20 μm. Since *E* = 1.6 × 10^11^ Pa and *ν* = 0.3, the spring constant of the torsion bar is given by *k_m_* = 6.7 × 10^–6^ + 2.5 × 10^–8^·*θ*^2^ (Nm) with the rotational angle *θ* in units of radians. 

Without applying a voltage to the electrostatic comb, the resonant frequency of scanner is given by the ratio of spring constant and inertia:

(17) $$f_{m0}=\frac{1}{2\pi }\sqrt{\frac{k_{m0}}{I_{\theta }}}$$

Therefore, the resonant frequency of the scanning micro-mirror with the application of a voltage to the electrostatic combs may be approximately obtained by introducing the total spring constant into Equation (17):

(18) $$\begin{array}{l} f_{me}=\frac{1}{2\pi }\sqrt{\frac{k_{m}+k_{e}}{I_{\theta }}}=\frac{1}{2\pi }\sqrt{\frac{k_{m0}+k_{e0}+(k_{m0}\alpha -k_{e0}\beta )\theta^{2}}{I_{\theta }}}=\frac{1}{2\pi }\sqrt{\frac{k_{m0}+k_{e0}}{I_{\theta }}}\times \sqrt{1+\frac{k_{m0}\alpha -k_{e0}\beta }{k_{m0}+k_{e0}}\theta^{2}}\\ \approx \frac{1}{2\pi }\sqrt{\frac{k_{m0}+k_{e0}}{I_{\theta }}}\left(1+\frac{1}{2}\times \frac{k_{m0}\alpha -k_{e0}\beta }{k_{m0}+k_{e0}}\theta^{2}\right)\; \end{array}$$

The frequency shift Δ*f_me_*_0_ by operating the electrostatic combs at a small angle is expressed by using the linear part of the spring constants of the torsion-bars and the electrostatic combs under the condition of *k_e_*_0_ << *k_m_*_0_ as:

(19) $$f_{me0}=f_{m0}+\Delta \;f_{me0}=\frac{1}{2\pi }\sqrt{\frac{k_{m0}+k_{e0}}{I_{\theta }}}=\frac{1}{2\pi }\sqrt{\frac{k_{m0}}{I_{\theta }}\left(1+\frac{k_{e0}}{k_{m0}}\right)}\approx f_{m0}\left(1+\frac{1}{2}\cdot \frac{k_{e0}}{k_{m0}}\right)$$

Therefore:

(20) $$\frac{\Delta \;f_{me0}}{f_{m0}}\approx \frac{1}{2}\cdot \frac{k_{e0}}{k_{m0}}$$

Moreover, when the rotation angle is large, the nonlinear coefficients influence the oscillation frequency. The excess increase in resonant frequency from the resonant frequency at the small angle is expressed by:

(21) $$f_{me}=f_{me0}+\Delta \;f_{me}(\theta )$$

The increased rate proportional to the square of *θ* can be defined as a nonlinear rate *R_S_* as follows:

(22) $$R_{S}=\frac{\Delta \;f_{me}}{f_{me0}}\approx \frac{1}{2}\cdot \frac{k_{m0}\alpha -k_{e0}\beta }{k_{m0}}\theta^{2}$$

The nonlinear rate *R_S_* corresponds to the bending of oscillation curve.

Under our designed conditions, the rotational inertia is given by *I_θ_* = 4.2 × 10^−16^ Nm and, thus, the calculated resonant frequency *f_m_*_0_ is approximately 29 kHz. From the designed parameters of the electrostatic combs, the values are given as, *l* = 300 μm, *a* = 100 μm, *b* = 20 μm, and *g* = 5 μm. The number of comb finger gaps is obtained from the four combs on the both sides and they are totally *N* = 80. Using the permittivity *ε*_0_ = 8.85 × 10^−12^ F/m, the spring constant of electrostatic combs is obtained analytically as *k_e_* = (2.6 × 10^–11^ − 8.5 × 10^–10^·*θ*^2^)*V*^2^. When the designed values are applied to Equations (20) and (22), we obtain:

(23) $$\frac{\Delta \;f_{me0}}{f_{me0}}\approx \frac{1}{2}\left(\frac{k_{e0}}{k_{m0}}\right)=\frac{1}{2}\cdot \frac{2.63\;\times \;10^{-11}V^{2}}{6.96\;\times \;10^{-6}}$$

(24) $$R_{S}=\frac{1}{2}\cdot \frac{2.49\;\times \;10^{-8}-8.47\;\times \;10^{-10}V^{2}}{6.96\;\times \;10^{-6}}\theta^{2}$$

From Equation (24), the comb voltage necessary for compensating the nonlinearity of the torsion-bar is estimated roughly to be 5.4 V from the condition of *R_S_* = 0.

## 3. Design and Fabrication

**Figure 5:**
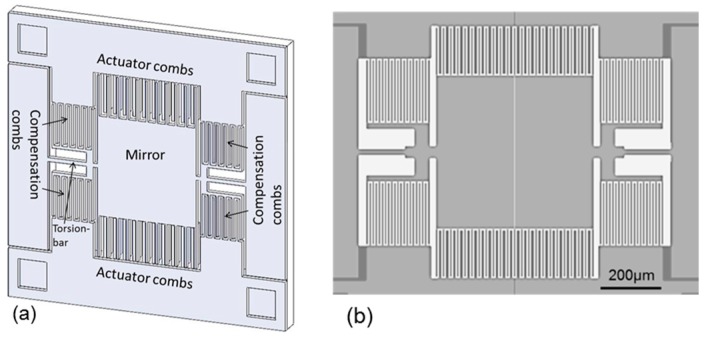
Schematic diagrams of scanner: (**a**) oblique view; and (**b**) top view.

Based on the principle described in Section 2, a one-dimensional scanning micro-mirror with the electrostatic compensation combs is designed and fabricated. Figure 5a shows the oblique schematic diagram of the scanning micro-mirror, which consists of a mirror plate, two torsion-bars, two pairs of actuator combs, and four pairs of the compensation combs. The mirror plate is 500 μm square and 20 μm in thickness, which is equal to the thickness of the top silicon layer of SOI wafer. The torsion-bars are 250 μm in length (symbol *L* in Section 2) and 20 μm in width and thickness (symbols *w* and *b*). Figure 5b shows the top view of the scanning micro-mirror. In order to rotate the micro-mirror, the vertical comb-drive actuators are installed at the edges of mirror plate as shown in Figure 5a. The movable fingers of the actuator combs are 200 μm in length, 5 μm in width, and 20 μm thick. The fixed fingers of the actuator combs are 200 μm in length, 8 μm in width, and 200 μm in thickness, which is same as thickness of the silicon substrate of SOI wafer. Therefore, the movable combs and the fixed combs are different in height, the former is on the top silicon layer of SOI wafer and the latter is on the silicon substrate. The height difference is 21 μm, which is equal to the addition of the thicknesses of the top silicon layer and the buried oxide layer of SOI wafer. The overlap length of the actuator comb fingers is 190 μm, and the gap between the movable and fixed fingers is 5 μm. The distance between the bottom of the movable fingers and the top of the fixed fingers of the actuator comb is 5 μm. The number of the finger gaps is 44 for each side of the micro mirror.

On the other hand, the compensation combs are located around the torsion-bars as shown in Figure 5. The movable and fixed fingers of the compensation combs are 205 μm in length, 5 μm in width, and 20 μm (symbol *b*) in thickness. The gap (*g*) between the movable and fixed comb fingers is 5 μm. The distance (*l*) from the rotation axis to the end of movable fingers is 300 μm, and the distance (*a*) from the rotation axis to the end of fixed fingers is 100 μm. The width of the compensation comb fingers is 5 μm. The number of the finger gaps of each compensation comb is 40. 

**Figure 6:**
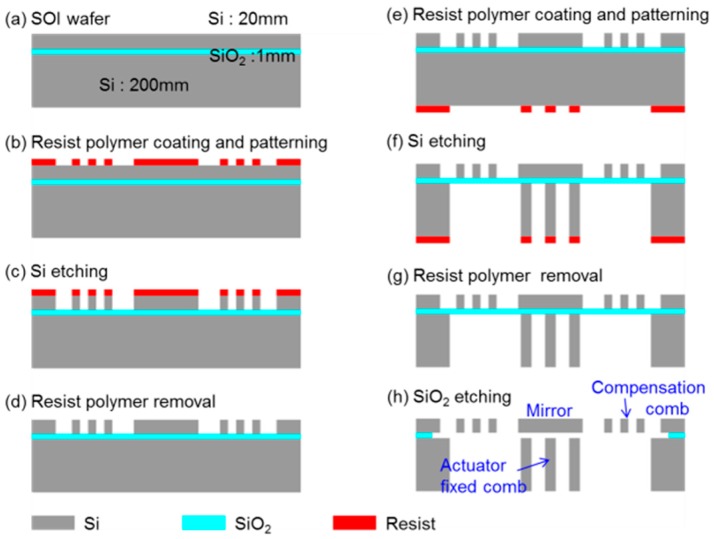
Schematic diagram of the fabrication processes.

The fabrication steps are shown in Figure 6. The SOI wafer used for the fabrication consists of a 20 μm thick top silicon layer, 1 μm thick buried oxide layer, and 200 μm thick silicon substrate. The top silicon layer is coated by a resist polymer (OFPR800-200cp, Tokyo Ohka. Kogyo Company, Ltd., Kawasaki, Japan) (a and b), and patterned by deep reactive ion etching (c). After removing the resist polymer (d), the back side of the wafer is coated by the resist polymer and patterned (e). The silicon substrate is etched from the backside by the deep reactive etching (f). After removing the resist polymer (g), the buried oxide layer is partially etched by a buffered hydrofluoric acid solution. Finally, the device is dried after replacing the acid solution with water, ethanol, and isopropyl alcohol to prevent comb fingers from sticking.

## 4. Fabrication Results and Operation Characteristics in Vacuum

**Figure 7:**
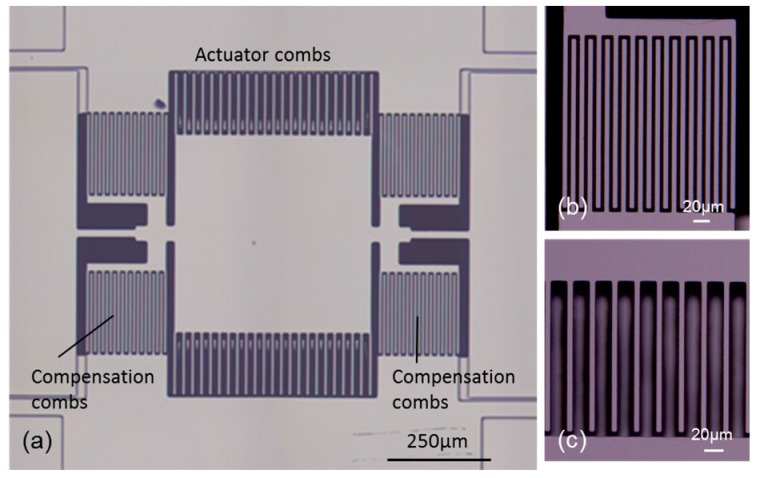
Optical micrograph of the fabricated scanning micro-mirror: (**a**) whole view; (**b**) compensation combs; and (**c**) actuator combs.

Figure 7a shows the whole view of the fabricated scanning micro-mirror. The compensation combs are fabricated on the same plane of the top silicon layer as shown in Figure 7b. Figure 7c shows the magnified view of the actuator combs, where the height difference between the movable comb (top silicon layer) and the fixed comb (silicon substrate) is seen from the defocused image of the lower comb. The mirror plate can be rotated around the silicon torsion-bars by the initial toque generated by the actuator combs having the height difference.

**Figure 8:**
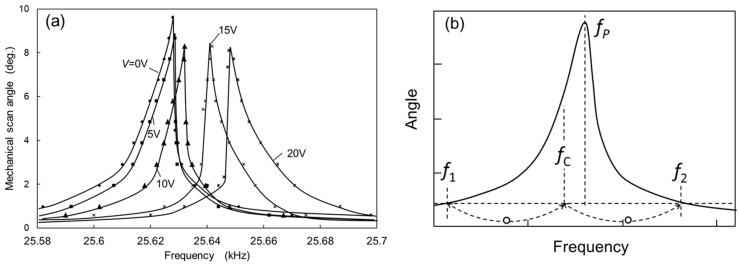
(**a**) Mechanical scan angle measured as a function of the frequency with the voltage *V* applied to the compensation combs as a parameter; and (**b**) the frequencies for obtaining the value of *R_S_*.

The mechanical scan angle was measured from the deflection angle (optical scan angle) of a laser beam impinging on the mirror plate. The deflection angle was obtained from the length of the laser scan line on screen and the distance between the mirror plate and the screen. The scanning micro-mirror was placed in a vacuum chamber and the air dumping effect was nearly removed at a pressure of 30 Pa. Figure 8a shows the mechanical scan angle (a half of the optical scan angle) measured as a function of the frequency of the applied voltage. The voltage (*E*(*t*)) applied to the actuator combs was an AC voltage of 10 V amplitude with 10 V DC voltage (*E*(*t*) = 10sin(2*πft*) + 10 *V*, *t*: time, *f*: frequency). Without applying DC voltage *V* for the compensation combs, the peak mechanical rotation angle *θ* is approximately 9.7 degrees at the frequency of 25.628 kHz. The rotation angle increases gradually with increase in the frequency of *E*(*t*) before reaching the peak amplitude, and rapidly decreases with the increase in the frequency after the peak amplitude. Since the decrease from the peak amplitude is steeper than that for the increase to the peak amplitude, the oscillation amplitude curve is not symmetrical with respect to the peak frequency (*f_P_*), and the peak frequency is shifted to higher frequency from the symmetrical position. This is mainly caused by a hard-spring effect of the torsion-bars. The quality factor of the oscillation can be roughly obtained from the full width at half maximum which is about 3000, although the oscillation curve is affected by the hard-spring effect.

In order to evaluate the nonlinearity of oscillation, the nonlinear rate *R_S_* can be approximately obtained from the measured oscillation curve as shown in Figure 8b. The nonlinear rate *R_S_* is nearly equal to the normalized frequency difference ((*f_P_* − *f_C_*)/*f_C_*) between the peak frequency (*f_P_*) and the center frequency (*f_C_*). The center frequency is obtained from the averaged frequency of the two frequencies (*f*_1_ and *f*_2_) where a horizontal line at a low angle level crosses the oscillation curve as shown in Figure 8b. Although the actual center frequency is the resonant frequency (*f_em_*_0_) corresponding to the peak frequency of the oscillation curve at a very small oscillation angle, we roughly estimate *f_em_*_0_ from *f_C_* assuming the symmetry of oscillation curve at the low angle level. The horizontal line at the low angle level is obtained around 10% of the peak angle. 

The peak frequency shifts to higher frequency by increasing the voltage *V* applied to the compensation combs. At the same time, the shape of the oscillation curve changes from the bend toward higher frequency to the bend toward lower frequency gradually with the increase of *V* from 0 to 20 V as shown in Figure 8a. The change of oscillation curve is caused by the change in the nonlinearity of the total spring by compensating the hard-spring effect of the torsion-bars with the soft-spring effect of the electrostatic spring. The peak frequency shift is caused by the increase in the linear part of spring constant by adding the electrostatic spring to the torsion-bar spring. 

**Figure 9:**
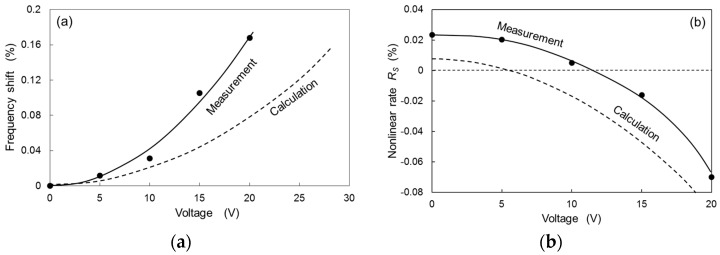
(**a**) Resonant frequency shift and (**b**) nonlinearity rate as a function of the voltage applied to compensation combs.

Figure 9a shows the resonant frequency shift normalized by the frequency at zero voltage (*V* = 0) as a function of the voltage applied to the compensation combs. The resonant frequency was roughly obtained from the center frequency (*f_C_*) as described above. The measured frequency shift varies nearly quadratically as a function of the applied voltage to the compensation combs. The measured frequency shift is roughly explained by the calculation using Equation (23), which is quadratically dependent on the applied voltage. 

Figure 9b shows the measured nonlinear rate *R_S_* with applied voltage, where *R_S_* defined as *R_S_* = (*f_P_* − *f_C_*)/*f_C_* (the frequencies are shown in the inset of Figure 8). The measured *R_S_* decreases with the increase in the voltage applied to compensation combs, and becomes zero at the voltage around 11.5 V. Therefore, by adjusting the applied voltage to the compensation combs, the nonlinearity of the torsion-bar spring can be compensated. The nonlinear rate calculated by Equation (24) is also shown in Figure 9b. The quadratic dependence of the measured *R_S_* is explained by the calculated *R_S_*, although the magnitude of the calculation is small compared to the measured value. The difference between the measurement and the calculation may be caused by the rough analytical calculation of the nonlinear rate of electrostatic combs. Since the actuator combs also generated electrostatic force, this may also cause additional nonlinear effect. Different actuation by piezoelectric or electromagnetic force can further clarify the difference between calculation and measurement.

## 5. Operation Characteristics in Atmospheric Air

The scanning micro-mirror was also operated in the atmospheric air and the operational characteristics were investigated. Compared to the operation in vacuum, a much higher voltage (~6 times) was needed for the operation of the actuator comb at the same oscillation angle. Large driving energy is required to overcome the air friction [4]. It was reasonable to consider that the actuator combs also generated some nonlinear effect since the torque was nonlinearly dependent on the rotation angle. The nonlinear effect of the actuator combs was also included in the measurement in vacuum, although the influence was smaller. The high voltage for the actuator combs seemed to cause a greater nonlinear effect, which might be proportional to the square of the AC voltage applied to the actuator combs. However, the analytical treatment of the nonlinearity of the actuator combs was more complex, since the torque was dependent not only on the rotation angle, but also on the alternating voltage. Here, although the analytical explanation cannot be provided, we describe only the operational characteristics and the compensation for the bending of oscillation curve by the application of a DC voltage to the compensation combs.

**Figure 10:**
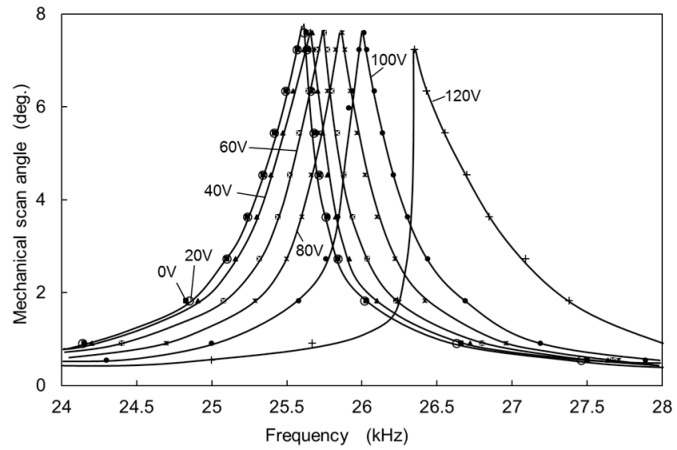
Mechanical scan angle measured as a function of the frequency with the voltage *V* applied to the compensation combs as a parameter.

Figure 10 shows the mechanical scan angles measured as a function of the frequency of the applied voltage to the actuator combs around the resonant frequency with the DC voltage applied to the compensation combs as a parameter. The voltage (*E*(*t*)) applied to the actuator combs to obtain nearly the same oscillation angle as in vacuum was *E*(*t*) = 60sin(2*πft*) + 60 *V*. The quality factor of the oscillation is obtained at about 53, which is smaller by a factor of 57 than that in vacuum. By increasing the voltage to compensation combs, the peak frequency shifts to higher frequency. The oscillation curve bends toward the higher frequency at the compensation voltage *V* ranging from 0 V to 60 V, and the bending is shifted towards the lower frequency at *V* from 100 V to 120 V as shown in Figure 10.

**Figure 11:**
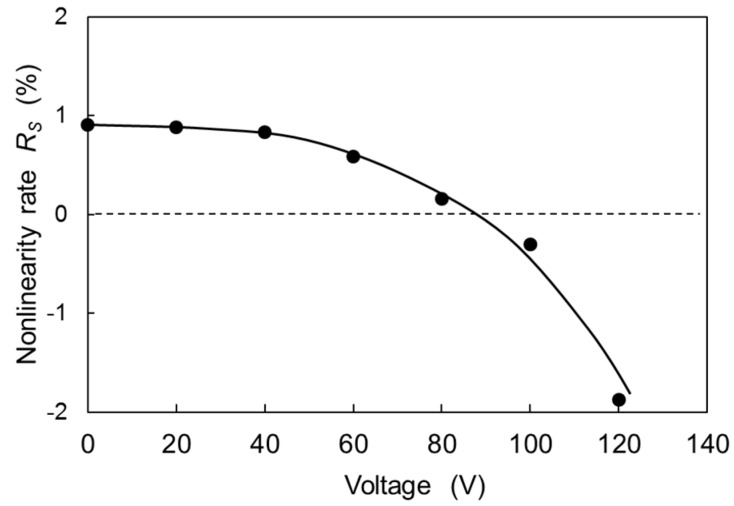
Nonlinear rate as a function of the voltage applied to the compensation combs.

Figure 11 shows the nonlinear rate *R_S_* measured as a function of the DC voltage *V* applied to the compensation combs. The value of *R_S_* is approximately 1% without applying *V*, which is larger than that of measured value in vacuum. Even if the value of *R_S_* is large, the nonlinearity can be compensated by increasing the DC voltage as shown in Figure 11. The nonlinear rate is nearly compensated at *V* of 87 V. Therefore, the proposed method is effective for compensating the nonlinear hard-spring effect caused not only by the hard-spring effect of the torsion-bar, but also by other effects. 

The proposed method can be applied to improve the control of scanning micro-mirror. When the scanning micro-mirror has such a large bend which causes amplitude jump in the oscillation curve [4], the operation frequency is usually adjusted to the frequency near the amplitude jump for maximizing the oscillation amplitude. However, the condition of the amplitude jump is likely to shift due to operation conditions, such as temperature. If the amplitude jump in the oscillation curve is removed by decreasing the hard-spring effect, the micro-mirror can be controlled more stably by maintaining the peak amplitude with a simple feedback loop.

## 6. Conclusions

The hard-spring nonlinearity of the torsion-bars of the scanning micro-mirror was compensated by a soft-spring effect of the electrostatic combs operated at DC voltage. The analytical model based on the capacitive electrostatic force was derived for explanation of the proposed method. The oscillation curve of the scanning micro-mirror suffering from the bend toward higher frequency was corrected to be nearly symmetric and further bent toward a lower frequency with the increase in the applied voltage to the compensation combs. In addition, the resonant frequency shifted by increasing the total spring constant. A 0.5 mm square scanner was fabricated from a 20 μm thick top silicon layer of a SOI wafer. The fabricated micro-mirror oscillated in vacuum at the resonant frequency around 26 kHz with a mechanical rotation angle of ±8 degrees. The nonlinear rate of 0.02% was compensated at the compensation voltage of 12 V in vacuum, which was roughly explained by the analytical calculation. Moreover, a 1% nonlinear rate at the atmospheric pressure was compensated at the voltage of 87 V. Therefore, the proposed method using the soft-spring effect of electrostatic combs was effective for the compensation of the hard-spring nonlinearity.
